# Cystic Nontubular Jejunal Duplication Cyst Presenting As Acute Intestinal Obstruction: A Surgical Challenge

**DOI:** 10.7759/cureus.13994

**Published:** 2021-03-19

**Authors:** Muhammad Khalid Syed, Ahmad A Al Faqeeh, Talal Almas, Hasan Alaeddin, Abdulla Hussain Al-Awaid

**Affiliations:** 1 Pediatric Surgery, King Fahad Hospital, Al Bahah, SAU; 2 Internal Medicine, Royal College of Surgeons in Ireland, Dublin, IRL

**Keywords:** intestinal duplication cyst, anastomosis, intestinal obstruction

## Abstract

Enteric duplication cysts are rare congenital anomalies that present with a vague constellation of symptoms such as vomiting and abdominal distension. Of these, cystic nontubular jejunal duplication cysts comprise an exceedingly small subset. Here, we delineate the case of a two-month-old female baby who presented with symptoms suggestive of acute intestinal obstruction. Radiological workup divulged a cystic lesion, which was subsequently confirmed to be a cystic nontubular jejunal duplication cyst with extensive intestinal wall sharing. Surgical excision was planned but posed a remarkable surgical challenge due to intestinal wall sharing and the cyst’s exceedingly fibrotic nature.

## Introduction

Gastrointestinal (GI) tract duplication cysts are rare congenital malformations that can occur anywhere within the GI tract. Their prevalence is noted to hover around one in 25,000 deliveries [1]. The duplication cysts can occur in the foregut, midgut, or hindgut. They are often diagnosed in the neonatal period, with computed tomography (CT) scan and endoscopic ultrasound (EUS) forming the cornerstone of diagnosis [1,2]. Cystic nontubular jejunal duplication cysts represent an exceedingly rare subset of these duplication cysts [3,4]. Clinically, jejunal duplication cysts present with a vague constellation of symptoms, including bilious vomiting, abdominal pain, bloating, and constipation, alluding to an underlying small bowel obstruction [5]. Here, we chronicle the case of a two-month-old girl who presented with a two-day history of bilious vomiting, constipation, and abdominal distension. Radiological investigation prompted suspicion of a cyst that was eventually confirmed to be a cystic nontubular jejunal duplication cyst upon exploratory laparotomy. Complete resection of the cyst was planned; however, due to the extensive wall sharing and the fibrotic nature of the cyst, the adjacent intestinal tissue had to be sacrificed, presenting a remarkable surgical challenge.

## Case presentation

A two-month-old female baby presented to the emergency department with a two-day history of bilious vomiting, constipation, and abdominal distension, suggesting possible intestinal obstruction. This presentation was on a background of intermittent episodes of bilious vomiting and a prior neonatal abdominal ultrasound remarkable for an underlying ovarian cyst, obscuring the current diagnosis. At the time of presentation, her symptoms included bilious vomiting, colic, constipation, and abdominal distension. Physical examination revealed a dehydrated and lethargic child who was underweight and visibly anemic. Pertinently, the abdomen was noted to be distended and a mass was palpable predominantly within the left hemiabdomen. The mass appeared mobile and nontender upon palpation. Based on the clinical picture and the patient’s past history of an ovarian cyst, a differential diagnosis including an ovarian cyst, duplication cyst, and mesenteric cyst was deemed plausible.

**Figure 1:**
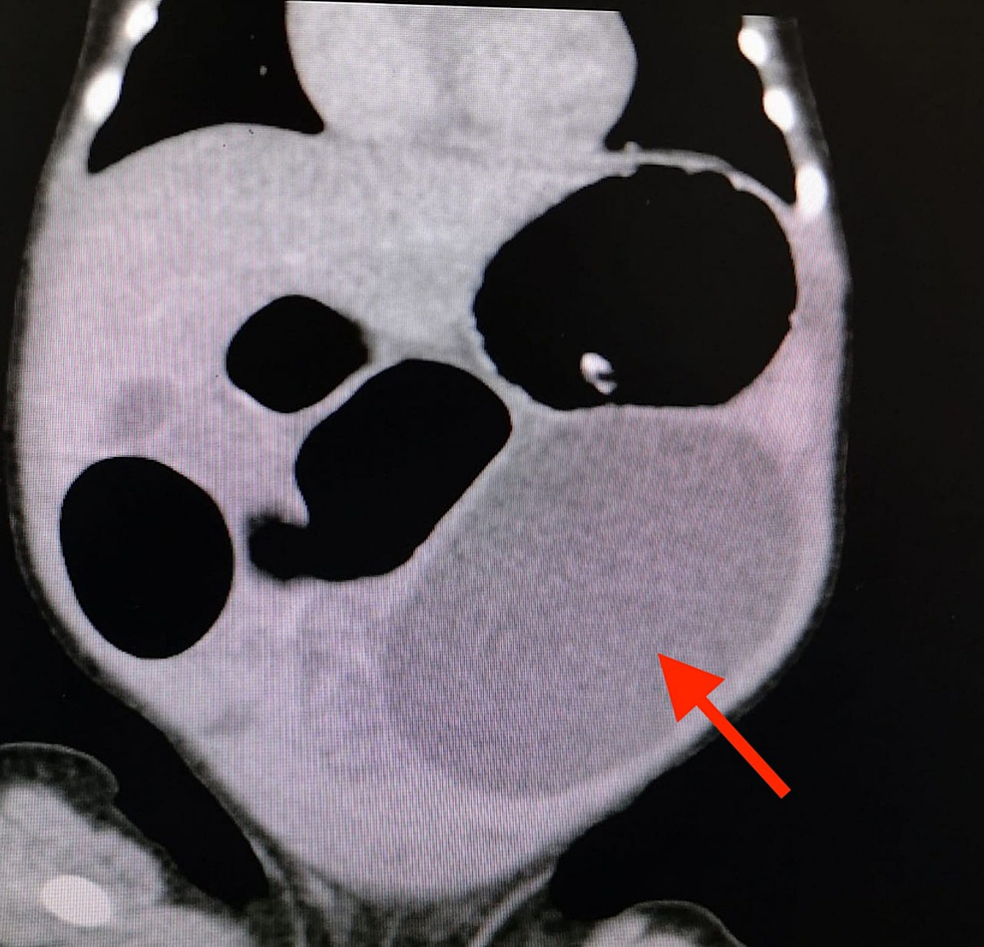
An abdominal CT scan showing a left-sided cystic lesion notably obstructing the small bowel (red arrow). CT, computed tomography

An abdominal radiograph was performed that showed a distended proximal bowel, with a generalized haziness in the abdomen. A subsequent CT scan revealed an exorbitant left-sided cystic lesion, obstructing the proximal intestine, the origin of which could not be ascertained (Figure 1).

**Figure 2:**
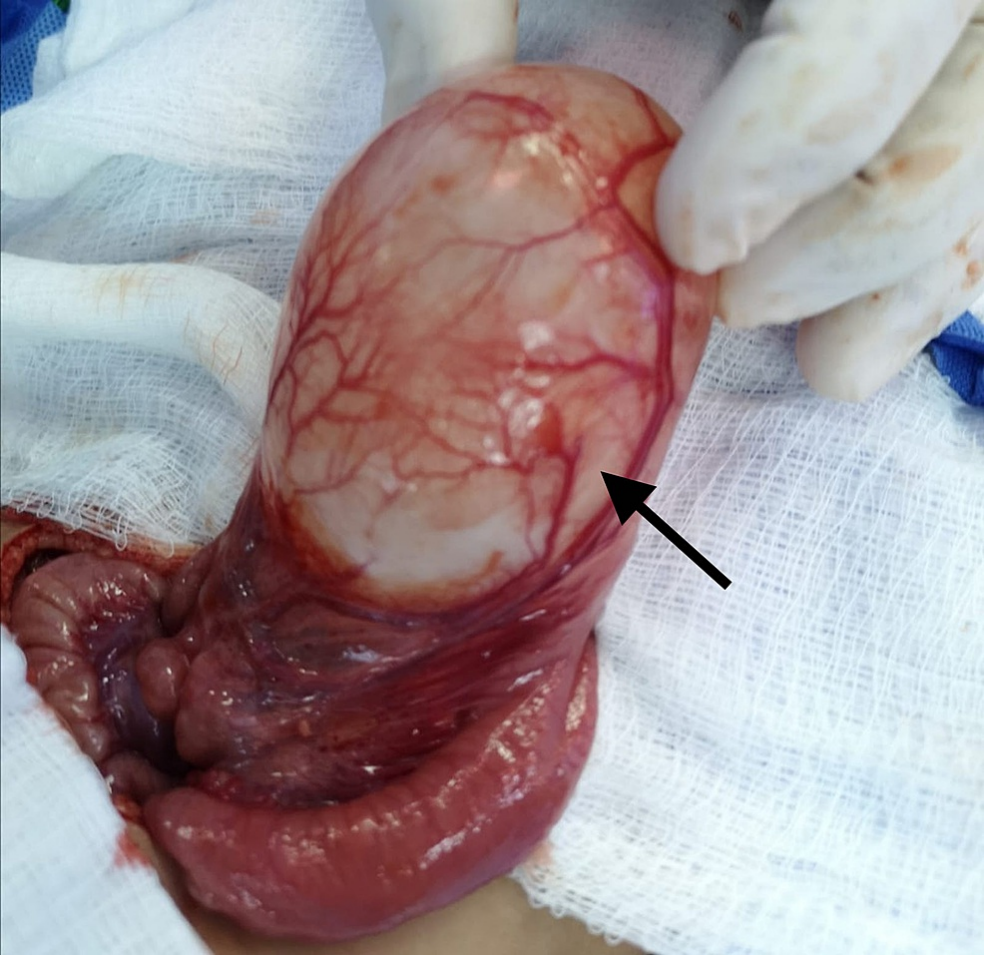
Intraoperative image showing the presence of a cystic lesion within the jejunal wall (black arrow).

The patient underwent resuscitation and blood transfusion, followed by an exploratory laparotomy performed through a left supraumbilical transverse incision. Upon laparotomy, a huge cystic lesion along the jejunal wall, about 25 cm from the duodeno-jejunal junction, was appreciated (Figure 2).

**Figure 3:**
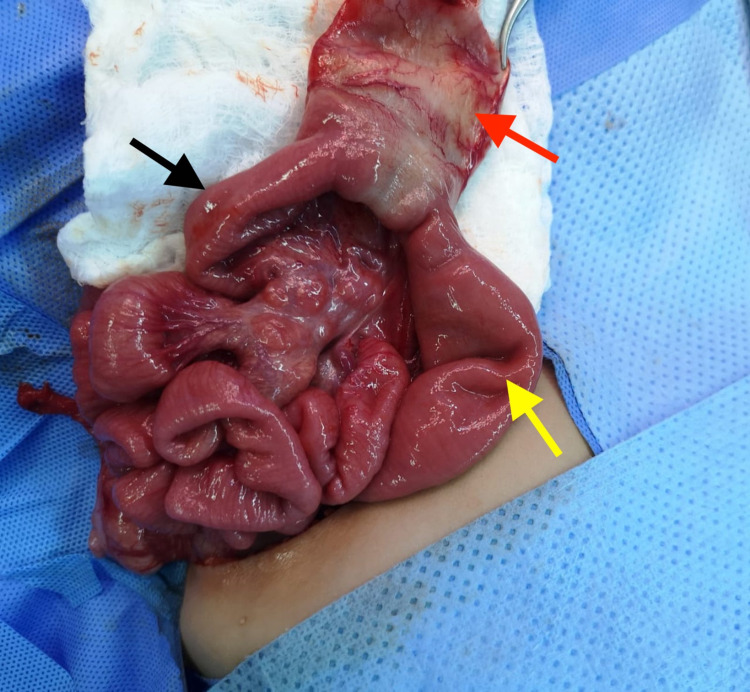
Intraoperative image showing a deflated jejunal cyst (red arrow), jejunum distal to the cyst (black arrow), and jejunum proximal to the cyst (yellow arrow).

Imperatively, the cyst was noted to share the jejunal wall, thereby causing jejunal obstruction and posing a remarkable surgical challenge as the adjacent wall had to be sacrificed. Therefore, an intraoperative diagnosis of a jejunal duplication cyst was made. Due to the extensive wall sharing and the fibrotic nature of the cyst, resection of the cyst and the adjacent jejunum was performed (Figure 3).

**Figure 4:**
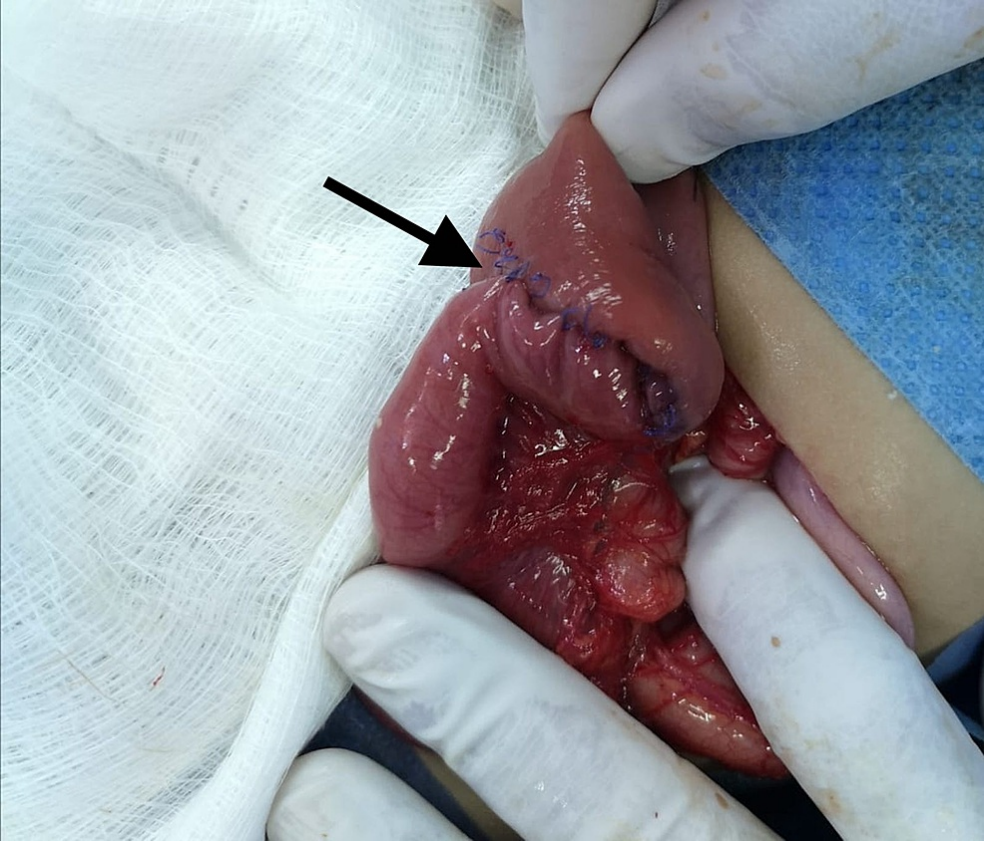
Intraoperative image demonstrating a jejunal side-to-side anastomosis (black arrow).

Thereafter, an anastomosis of the jejunum was performed simultaneously using a longitudinal GI anastomosis stapling device (Figure 4).

The baby demonstrated a smooth postoperative recovery. She was kept on total parenteral nutrition for four days, after which the transition to oral nutrition was made. Her bowel function remained intact. She was discharged on the seventh postoperative day and continues to do well to date.

## Discussion

GI tract obstruction remains one of the most frequent causes of postnatal admission to pediatric surgery units, with the most common causes being anorectal malformations, esophageal obstruction, and duodenal obstruction, with incidence rates hovering at 41%, 24%, and 20%, respectively [6]. A myriad of underlying etiologies can culminate in intestinal obstruction. These conditions include, among others, enteric duplication cysts, malrotation of the gut, meconium ileus, and annular pancreas [7,8]. The etiology of jejunal duplication cysts, like other enteric duplication cysts, remains elusive. Jejunal duplication cysts frequently present neonatally or before two years of age [8]. The most common clinical presentations of jejunal duplication cysts include signs and symptoms of small bowel obstruction, including vomiting, abdominal pain, bloating, constipation, and abdominal masses [5,9].

While the pathophysiology underlying jejunal duplication cysts remains esoteric, it is generally thought that these cysts represent a structure closely tethered to the jejunum with surrounding smooth muscle and an epithelial lining [10]. Due to its potentially grave clinical ramifications, prompt clinical evaluation and diagnosis in neonates presenting with the aforementioned symptoms is imperative. Interestingly, symptomatic jejunal duplication cysts are often diagnosed neonatally, whereas asymptomatic jejunal duplication cysts can often persist into adulthood and are rarely detected on incidental radiological imaging [3].

EUS is the diagnostic tool of choice for the investigation of all duplication cysts, and in some cases, is combined with fine needle aspiration of the cyst to obtain a definitive diagnosis and rule out other more serious pathologies, including potential underlying malignancies [4]. Other diagnostic modalities include plain radiograph of the abdomen, CT, and magnetic resonance imaging, which can all aid in the diagnosis and localization of the cyst [11]. The treatment of jejunal duplication cysts is mainly surgical and entails complete excision and curation of a primary anastomosis [9].

In our case, the jejunal cyst was noted to share a common intestinal wall, rendering it a considerable surgical challenge to safely resect the cyst alone. Furthermore, upon deflation, the cyst appeared exceedingly fibrotic, necessitating the excision of the adjacent jejunal wall. A complete resection and side-to-side anastomosis was thus performed. As duplication cysts amplify the chances of late malignant transformation, complete excision should be ensured [10]. However, in cases such as ours, excising the cyst alone without the adjacent intestinal tissue presents an insurmountable surgical challenge that surgeons must overcome to circumvent further episodes of acute obstruction. Imperatively, the diagnosis of a jejunal duplication cyst requires a laparotomy, and such complications are often appreciated intraoperatively at the time of surgery, further complicating the surgical case.

## Conclusions

Cystic nontubular jejunal duplication cysts are an exceedingly rare subdivision of enteric duplication cysts and can sometimes share a common wall within the GI tract. In such instances, complete resection can be a considerable surgical challenge, often necessitating the excision of the adjacent intestinal tissue. Due to their malignant potential and symptomatic presentation, complete excision remains necessary but exceedingly difficult in the context of preserving normal bowel function.
